# CCpos: WiFi Fingerprint Indoor Positioning System Based on CDAE-CNN

**DOI:** 10.3390/s21041114

**Published:** 2021-02-05

**Authors:** Feng Qin, Tao Zuo, Xing Wang

**Affiliations:** 1 College of Information Science and Engineering, Wuhan University of Science and Technology, Wuhan 430081, China; QinF7373@163.com; 2 Engineering Research Center for Metallurgical Automation and Detecting Technology of Ministry of Education, Wuhan University of Science and Technology, Wuhan 430081, China; wangxing199613@163.com

**Keywords:** WiFi fingerprint positioning, convolutional denoising autoencoder, convolutional neural network, K-means

## Abstract

WiFi is widely used for indoor positioning because of its advantages such as long transmission distance and ease of use indoors. To improve the accuracy and robustness of indoor WiFi fingerprint localization technology, this paper proposes a positioning system CCPos (CADE-CNN Positioning), which is based on a convolutional denoising autoencoder (CDAE) and a convolutional neural network (CNN). In the offline stage, this system applies the K-means algorithm to extract the validation set from the all-training set. In the online stage, the RSSI is first denoised and key features are extracted by the CDAE. Then the location estimation is output by the CNN. In this paper, the Alcala Tutorial 2017 dataset and UJIIndoorLoc are adopted to verify the performance of the CCpos system. The experimental results show that our system has excellent noise immunity and generalization performance. The mean positioning errors on the Alcala Tutorial 2017 dataset and the UJIIndoorLoc are 1.05 m and 12.4 m, respectively.

## 1. Introduction

In recent years, with the rapid development of the Internet of Things and mobile Internet, as well as the emergence of various wearable devices such as phone watches, indoor positioning has become one of the research hotspots in the field of location services and other major fields. Indoor positioning technology mainly includes Bluetooth [1], WiFi [2], radio frequency identification (RFID) [3], ultra-wide band (UWB) [3], etc. According to the different positioning principles, it can be divided into infrastructure-based positioning technology and infrastructure-less positioning technology. 

Infrastructure-based positioning technology is to deploy some auxiliary devices in the indoor environment. After the positioning device receives the signal strength from the auxiliary devices, the positioning device converts the signal strength into distance and then performs the positioning calculation through some algorithms. According to this, an indoor positioning algorithm based on Bluetooth low energy beacon [1] was proposed. In the framework of fuzzy logic, this algorithm used the received signal strength indicator (RSSI) of a Bluetooth low energy (BLE) beacon and the geometric distance from the current beacon to the fingerprint point to calculate the Euclidean distance for subsequent position determination. Infrastructure-free positioning technology involves installing prefabricated sensors in devices and combining knowledge of the environment to track location, including things like pre-existing Wi-Fi hotspots at sites. Orujov et al. [4] introduced the experimental study of indoor positioning algorithm based on the signal strength received from BLE beacons, proposing and implementing a scheme based on fuzzy logic. The most appropriate algorithm was selected according to the size of the room, the number of available beacons and the signal strength. Loizos kanaris et al. [5] combined BLE and WiFi for positioning, and filtered the initial fingerprint data set after measuring the proximity of RSS fingerprint to BLE devices through a new positioning algorithm, *i*-KNN, to achieve rapid positioning. As a large number of WiFi nodes are arranged in many places, and almost all mobile smart terminals have their own transceiver modules of WiFi signals, indoor positioning technology based on WiFi has natural advantages.

Indoor positioning technology based on WiFi can be mainly divided into two categories: the range-based localization algorithms and fingerprint-based localization algorithms [6].Among the range-based localization algorithms, the main algorithms include trilateral and triangulation positioning, which rely on the corresponding algorithms of time of arrival (TOA) [7], angle of arrival (AOA) [8], time difference of arrival (TDOA) [9], and receiving signal strength [10] to obtain the necessary distance and angle information. This kind of method is a positioning technology based on infrastructure, which requires special hardware equipment. In order to achieve high positioning accuracy, the position information of Access Point (AP) needs to be known in advance, which leads to its high cost and difficult to be widely used. However, the positioning technology based on location fingerprint has attracted widespread attention, because it makes full use of the extensive layout of WiFi nodes and mobile device resources without other hardware facilities.

**Figure 1:**
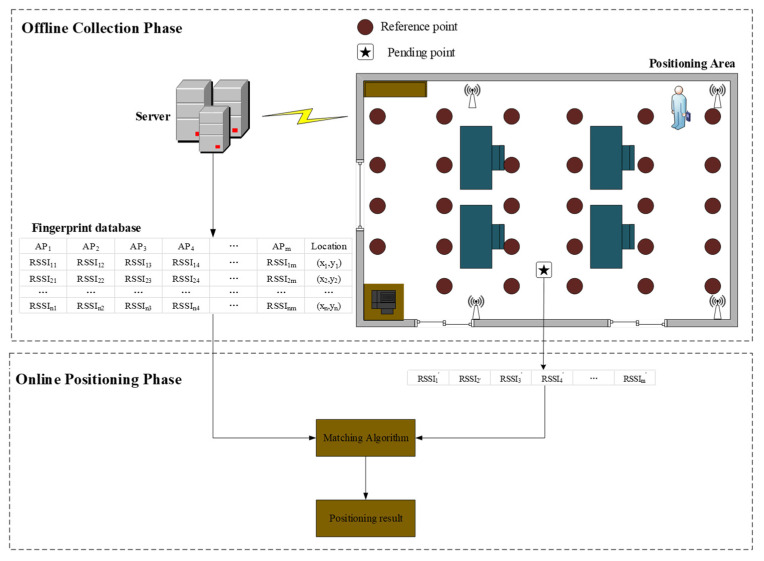
WiFi fingerprint positioning system.

The fingerprint-based positioning method consists of two stages: offline collection and online positioning [11]. In Figure 1, a certain number of reference points are collected in the positioning area. The main purpose of offline collection phase is to establish a location fingerprint database for representing the AP signal strength characteristics at each reference point in the location area. The fingerprint database is collected by mobile devices at reference points, including reference point coordinates and WiFi signal strength received by mobile devices at reference points. The purpose of the online positioning stage is to obtain the location information of the final positioning of the mobile terminal equipment. Within the positioning area, the mobile terminal equipment is utilized to detect the AP signal strength information received at the point, and the location fingerprint database generated in the offline training stage is used for matching calculation to obtain the location information.

Fingerprint-based positioning technology does not need to add other sensors to the environment. However, the RSSI value is unstable due to environmental interference, which results in low positioning accuracy. Duong Bao Ninh et al. [12] proposed to use random statistics to process the noise of WiFi signals and normalize the RSSI value when establishing an offline fingerprint database. In the online positioning stage, Mahalanobis distance was used to replace Euclidean distance for positioning to improve positioning accuracy. In the field of robot positioning, Le Zhang et al. [13] designed a lightweight indoor robot localization system that operates based on low-cost WiFi received signal strength (RSS) and can be readily plugged into any existing WiFi network infrastructure. And an end-to-end deep fuzzy forest algorithm was proposed for robust position estimation. The WiFi signal strength data collected during the algorithm learning phase is used to generate a perceptual model of the robot’s position assumptions, and the robot used the perceptual model to navigate autonomously in the indoor environment. Using WiFi positioning individually tends to cause fluctuations in positioning accuracy, thus many scholars have started to research positioning technologies that integrate WiFi with other sensors or use other algorithms to improve WiFi positioning. Myungjun Jin et al. [14] presented an IMU (Inertial Measurement Unit)-assisted fingerprint positioning nearest neighbor selection algorithm, which used the location prediction of IMU measurement values to filter out irrelevant reference points, thereby reducing the positioning error from the RSS variation problem. HaiFeng Yang et al. [15] proposed a weighted K-nearest neighbor (WKNN) indoor localization algorithm based on spatial feature partitioning and former position restriction. In this system, a large target space is partitioned into multiple partitions based on spatial features, while a finite relationship between the former and the present position is introduced to improve the quality of the selected candidate reference points, thus significantly improving the smoothness of the estimation results.

Location fingerprint positioning technology has been relatively mature, and it is a positioning technology with good civilian and commercial prospects. However, there are still shortcomings and many problems that have not been resolved. The main reason lies in the presence of obstacles in the indoor environment, changes in natural conditions, and movement of people. The existing techniques, such as, K-nearest neighbor (KNN) [16], WKNN [17] and support vector machine (SVM) [18], are complicated and easily interfered, so the positioning performance cannot meet the practical requirements. With the successful application of deep learning in images, recurrent neural networks (RNN) [19] and deep neural network (DNN) [20] have been applied in the field of localization. However, the RNN-based methods need to collect sufficient time series data, and the method based on DNN has high computational complexity and the phenomenon of parameter inflation. This paper proposes a positioning system using CDAE and CNN, and utilizes K-means clustering algorithm to partition the data set in the offline stage, so that this positioning system has high localization accuracy even on small datasets. Compared with existing indoor localization methods, the main contributions of this paper are as follows:

(a) Our system adopts K-means clustering algorithm to extract the validation set from the All-Training set in the offline fingerprint database. It solves the problem that the random selection method may lead to fluctuations in localization due to incomplete data coverage of the training set in the case of small data sets.
(b) This system designs a new network model that combines CDAE and CNN. This model utilizes the CDAE network to reduce the data dimensionality, while using the training process of adding noise and performing noise reduction to force the network to learn more robust invariant features and obtain a more effective representation of the input. Additionally, key features can be extracted from the RSSI data and then the CNN is trained to achieve high success rate effectively in the localization phase.

The rest of this paper is organized as follows. We review related work on deep learning in WiFi fingerprint localization in Section 2, and Section 3 presents the architecture of the CDAE-CNN based system proposed in this paper, including an overall overview of the system and an introduction to the system architecture. Section 4 optimizes our system model through experiments and compares it with other fingerprint-based localization algorithms. Section 5 summarizes the work of this paper and the outlook of future work.

## 2. Related Work

Hinton’s research group participated in the ImageNet Large-Scale Visual Recognition Challenge and won the championship through the constructed CNN network AlexNet, and crushed the classification performance of the second place (SVM) in 2012. Since then, CNN has attracted the attention of many researchers in the image field. With the continuous advancement of network structure, training methods, and graphic processing unit (GPU) hardware, it continues to conquer the battlefield in other fields. Mai Ibrahim et al. in [21] proposed an indoor localization method based on CNN. By using RSS time-series of wireless local area network access point to locate, the noise and randomness of individual RSS values would be reduced to improve the localization accuracy. Jin-woo Jang and Song-Nam Hong [22] used a convolutional neural network to train the topology and signal strength of a radio map, which is robust to small changes in the received signal. However, the network of this method is prone to overfitting phenomenon and is just a simple CNN classification network structure. In order to extract more representative features from RSSI data, Xudong Song et al. [23] employed stacked auto-encoder to extract major features from RSS data, and then used CNN training data. The method achieves good results in floor classification, but has a large error in the specific location, and the method is also susceptible to RSSI fluctuations. As other network structures have also developed, Weizhu Qian et al. [24] put forward a network model combining CNN, RNN, and mixture density networks (MDN). The CNN sub-model is for detecting the characteristics of high-dimensional input. The RNN sub-model is leveraged to capture the time dependence, and the MDN sub-model is employed to predict the final output. Jing Zou et al. [25] utilized DNN and CNN to predict user location, respectively, and then exploited the Dempster–Shafer (DS) algorithm to fuse the results of two network structures to get the final result. Both of them use CNN and DNN models to predict the user’s location separately and then use a fusion algorithm to get the final location. This will lead to high time complexity and long execution time of the localization method.

For convolutional neural networks, the number of data sets used for learning is the most important factor in achieving high accuracy. Due to the high cost of collecting data, it is necessary to improve the accuracy on small data sets. The above articles work well in floor and building positioning, but the error in the specific location is very large. In this paper, we use the K-means algorithm to segment the dataset and then use the CDAE-CNN network for location prediction. This will achieve more accurate and efficient positioning. In addition, this method requires less dataset. And the system has faster execution time.

## 3. System Overview

This section first introduces the overall architecture of the system, and then describes each part of the system separately. The system includes data preprocessing, validation set from the all-training set with the K-means algorithm and introduction of the structure of the location estimation model.

**Figure 2:**
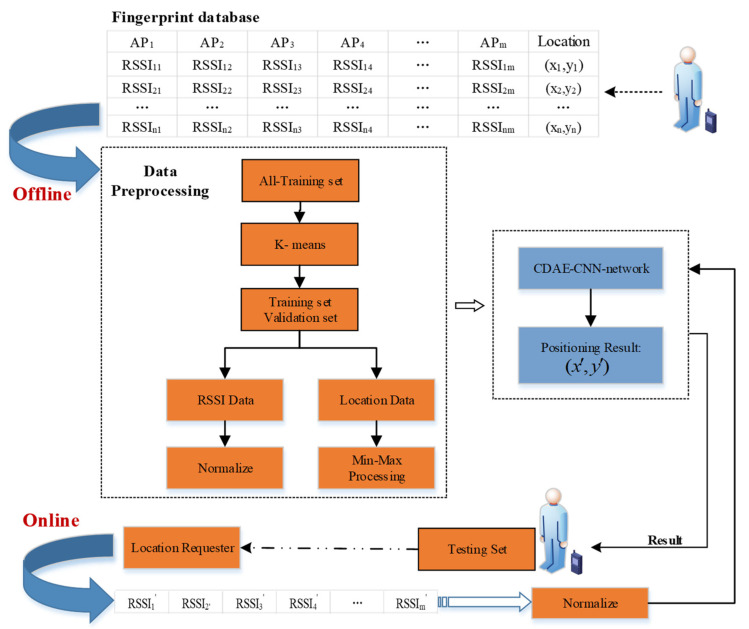
System architecture of CCpos.

Figure 2 shows the system architecture of CCpos. The system is divided into offline data preprocessing stage and online location prediction stage. In the offline stage, the information of reference points in the undetermined area is collected to establish the offline fingerprint database. Then the all-training set and the testing set are divided according to the ratio, generally 4:1. The verification set and training set are extracted from the all-training set data by the K-means algorithm. Each data sample is divided into RSSI data and location data by processing them with different formulas to map them between 0 and 1. Subsequently the processed data is input into CDAE and CNN network structures for training. In the online location prediction stage, the user first sends the location request, and then inputs the RSSI data obtained by the user’s mobile device into the CCpos system after normalization processing. The system will send the location result to the requester. In this paper, in order to obtain the positioning error of the system, the testing set is taken as the real-time positioning data of the user and input into the system. 

How to use the K-means algorithm to extract validation sets, the data normalization process, and the network structure for location prediction are described in detail in the following sections

### 3.1. Extract Validation Sets from the All-Training Set—K-Means

In machine learning, it is necessary to adjust the parameters of the model when developing it, such as changing the weights and the size of each layer, or selecting the number of layers. This adjustment process needs to provide a feedback signal from the performance of the validation set data on the trained model to modify the network model and parameters. Therefore, the validation set is important in the training process. Nowadays, the common method for extracting the validation set is random extraction, which leads to the fact that the validation set may not contain sample points for each region in the localization area. To solve this problem, we propose to extract the validation set from the all-training set using the K-means algorithm [26].

Algorithm 1 presents how to extract the validation set using the K-means algorithm. The inputs on the algorithm are all-training sets AT and the number of clustering centers *K*. The outputs are the training set T and the validation set V.

First, based on the two columns of data representing the coordinates in the all-training set AT, *K* cluster centroids are randomly selected: (*x*_1_, *y*_1_),…, (*x_k_*, *y_k_*) and *K* clustering centers constitute the set KC. We calculate which class each sample belongs to and then recalculate the clustering center for each class. Repeat this process until the clustering centers are unchanged. The final set KC is obtained, and the clustering center is $\left(x_{1}^{\prime },y_{1}^{\prime }\right),\cdots ,\left(x_{k}^{\prime },y_{k}^{\prime }\right)$. For each cluster center, find the nearest sample point and put it into the validation set V. The other sample sets are put into the training set T.
**Algorithm 1.** K-means extraction of validation sets**Input**: All-training set data AT. Number of clustering centers k**Output**: Training set T. Verification set V. 1: According to the two columns of data representing coordinates in All-Training sets AT, k cluster centers are randomly selected: $\left(x_{1},y_{1}\right),\cdots ,\left(x_{k},y_{k}\right)$. Define an empty list KC. 2: Put the point $\left(x_{j},y_{j}\right)$ into KC, where j∈(1,k). 3: **for**
$\left(x_{i},y_{i}\right)$
**to** the last two columns of data in AT do 4:   **for**
$\left(x_{j},y_{j}\right)$
**to** KC **do** 5:      Repeat the process until the clustering center remains unchanged  6:         { 7:           //For each example, calculate the class it should belong to. 8:          $c^{\left(i\right)}:=\arg\;\underset{j}{min}{\left\|(x_{i},y_{i})-(x_{j},y_{j})\right\|}^{2}$ 9:         //For each class, recalculate its cluster center instead of the cluster center at the original location. 10:         $\left(x_{j}^{\prime },y_{j}^{\prime }\right):=\frac{\Sigma_{n}^{i=1}\;1{\left\{c^{\left(i\right)}=j\right\}}^{\left(i\right)}}{\Sigma_{n}^{i=1}\;1\left\{c^{\left(i\right)}=j\right\}}$ 11:         Put the point $\left(x_{j}^{\prime },y_{j}^{\prime }\right)$ into KC. 12:       } 13:    **end for** 14: **end for** 15://The sample points closest to the cluster center are placed in the validation set V. 16: $I=\arg\;\underset{i}{min}\sqrt{{\left(x_{i}-x_{j}^{\prime }\right)}^{2}+{\left(y_{i}-y_{j}^{\prime }\right)}^{2}}$ 17: Put the data in row *I* of AT into V.  18: Delete row *I* data from AT.  19: T←AT  20: **return** T, V 

### 3.2. Data Preprocessing—Normalization

This subsection describes the process of normalizing the RSSI and coordinate values of the dataset. Converting the RSSI and coordinates to a range of 0–1 is useful for increasing the learning ability of the neural network model. The coordinates are converted to a range of 0–1 with Equation (1), and after training, the coordinates are restored to their original values with Equation (2), where ${\left(x,y\right)}_{min}$ and ${\left(x,y\right)}_{max}$ are the minimum and maximum values for all coordinates. The paper [27] presents three normalization methods for RSSI: zero-to-one normalized representation, exponential representation and the powed representation. Different representations of RSSI will lead to different accuracy of positioning results. The three representations are shown in Equations (3)–(5), where *i* represents the WAP identifier, ${RSSI}_{i}$ is the received signal strength indication of the *i*-th WAP, and *min* is the minimum value of RSSI in the offline fingerprint database. The parameters *α* represent the mathematical constant *e*, whose value is about 2.7. Our paper compares the experimental results of the three methods separately, where the mean positioning error obtained by the zero-to-one normalized representation is the smallest.

(1) $$\left(x_{n}^{\prime },y_{n}^{\prime }\right)=\frac{\left(x_{n},y_{n}\right)-{\left(x,y\right)}_{min}}{{\left(x,y\right)}_{max}-{\left(x,y\right)}_{min}}$$

(2) $$\;\left(x_{n},y_{n}\right)=\left(x_{n}^{\prime },y_{n}^{\prime }\right)\times \left[{\left(x,y\right)}_{max}-{\left(x,y\right)}_{min}\right]+{\left(x,y\right)}_{min}$$

(3) $${\mathrm{ZeroToOneNormalized}}_{i}=\{\begin{array}{cc} 0, & RSSI_{i}=100\\ \frac{RSSI_{i}-min}{-min}, & -99\leq RSSI_{i}\leq 0 \end{array}$$

(4) $${\;\mathrm{Exponential}\;}_{i}=\{\begin{array}{cc} 0, & RSSI_{i}=100\\ \frac{\exp\left(\frac{RSSI_{i}-min}{\alpha }\right)}{\exp\left(\frac{-min}{\alpha }\right)}, & -99\leq RSSI_{i}\leq 0 \end{array}\;$$

(5) $${\mathrm{Powed}}_{i}=\{\begin{array}{cc} 0, & RSSI_{i}=100\\ \frac{{\left(RSSI_{i}-min\right)}^{\alpha }}{{\left(-min\right)}^{\alpha }} & -99\leq RSSI_{i}\leq 0 \end{array}\;$$

### 3.3. Position Estimation Model

**Figure 3:**
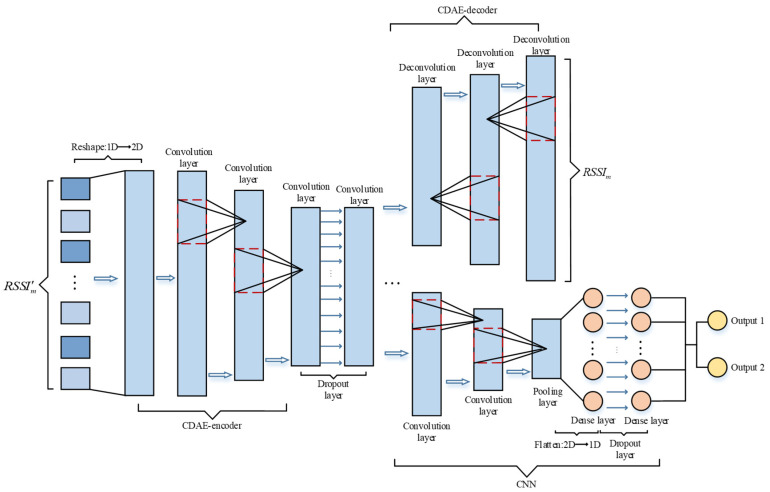
CDAE-CNN network structure.

As shown in Figure 3, the position estimation model CDAE-CNN consists of a convolutional denoising autoencoder and a convolutional neural network. The input RSSI data is first transformed from one-dimensional to two-dimensional and then fed into the CDAE. The convolutional denoising autoencoder [28] can help the model extract useful features from the data and improve the noise immunity of the model, and the computation amount is lower than that of other denoising autoencoders.

For each fingerprint point, the RSSI data is first injected with Gaussian white noise (GWN) n(t)  with a mean of 0 and variance of 1, where the intensity of the noise is controlled by the noise coefficient λ. The original data and the corrupted noise data are then used as inputs to the CDAE, which helps to reduce model overfitting by forcing the model to learn the inherent characteristics of the data. We can obtain the corrupted RSSI data as given in Equation (6):

(6) $$RSSI^{\prime }=RSSI+\lambda *n\left(t\right)$$

CDAE contains an encoding part and a decoding part. The encoding part extracts the robustness characteristics of the data RSSI’ injected with noise, which is mainly convolution operation. The decoding part is the process of reconstructing the obtained RSSI, which is mainly a deconvolution operation. The purpose of the deconvolution operation is to increase the dimensionality of the input features, which is calculated in the same way as the convolution operation. The mapping relationship between the broken (noise added) input and the pure output can be obtained after CDAE training.

After the RSSI data features are extracted from the coding part of the CDAE, they pass through a dropout layer and then transmit to the CNN network. The number of neurons connected to the CNN network is controlled by adjusting the size of the dropout rate to reduce the model parameters. The CNN network predicts the user’s location and includes convolutional layers, pooling layers and dense layers. After extracting the features through the convolutional and pooling layers, the two-dimensional data is flattened into one-dimensional data, and then the results are output through the fully-connected layer. Outputs 1 and 2 are the values of coordinates x and y, respectively. The convolutional neural networks used in this system are all one-dimensional convolutional neural networks. The specific number of convolutional layers will be introduced in the experimental section, and the number of convolutional layers to obtain the optimal localization accuracy is different for different datasets. In order to prevent overfitting, a dropout layer is also added in front of the dense layer.

In our system, in addition to using additional dropout layers to reduce overfitting, we also use the early stopping method. The early stopping method is used to calculate the performance of the model on the validation set during training. When the performance starts to decline, the training will stop, and the parameters from the previous iteration results serve as the final parameters of the model. In this paper, we choose to monitor the error of the validation set and use the patience parameter *X* to control the training process. If the error of the validation set does not decrease in *X* iterations, then training is stopped.

## 4. Evaluation

In this section, we evaluate the performance of the CCpos system on two publicly available datasets: UJIIndoorLoc [29] and the Alcala Tutorial 2017 dataset [30]. First, we describe the two datasets and the model’s inputs and outputs. Then, we optimize our system model through experiments. Finally, we compare the experimental results obtained by our system with the results obtained by other WiFi-based fingerprinting methods. We use Python-3.6.12, Keras-2.3.1, and Tensorflow-1.14.0 to train the CCpos system. 

### 4.1. Dataset

This paper employs a large data Set (UJIIndoorLoc) and a small dataset (Alcala Tutorial 2017 Data Set). UJIIndoorLoc is the largest open access indoor location database, containing 21,049 fingerprint samples, covering three buildings of 4–5 floors. In addition, there are 520 APs within the positioning range. The tag contains (x,y,f,b), indicating that the reference point (x,y) is located on the floor *f* of building *b*. However, the main consideration of our system is location positioning. For UJIIndoorLoc data set, the input of the system model is $\vec{r}=\left(r_{1},r_{2},\cdots ,r_{520}\right)$, and the output is (x,y,f,b). The Alcala Tutorial 2017 dataset mainly studies the small scene in the corridor of the Engineering of the University of Alcala (Spain), which is another option of the UJIIndoorLoc dataset, including 152 WiFi fingerprint signal strength data and coordinates of reference points. Alcala Tutorial 2017 dataset is composed of 670 training sets and 405 test sets. Therefore, for Alcala Tutorial 2017 dataset, the input of CCpos system is $\vec{r}=\left(r_{1},r_{2},\cdots ,r_{152}\right)$ and the output is (x,y). The WiFi signal strength value of the two data sets is expressed as the negative integer values from −99dBm to 0dBm, and the value of 100 is used to indicate that no WAP was detected. 

### 4.2. Evaluate Experimental of Alcala Tutorial 2017 Dataset

This subsection presents the optimization of the system model based on Alcala Tutorial 2017 dataset and the comparison of experimental results with other WiFi fingerprint-based methods.

The parameters used to optimize the model in the system are given in Table 1. In CDAE and CNN models, the activation functions and output layer are all rectified linear units (ReLUs). The optimizer is Nadam. The loss function is the mean square error (MSE), and the monitoring index is the mean absolute error (MAE). The training batch is set to 18.

#### 4.2.1. CDAE-CNN Model Optimization

This section describes the optimization process of the CDAE-CNN model, including the selection of the network structure, the selection of the noise coefficient, and the selection of the dropout rate.

**Figure 4:**
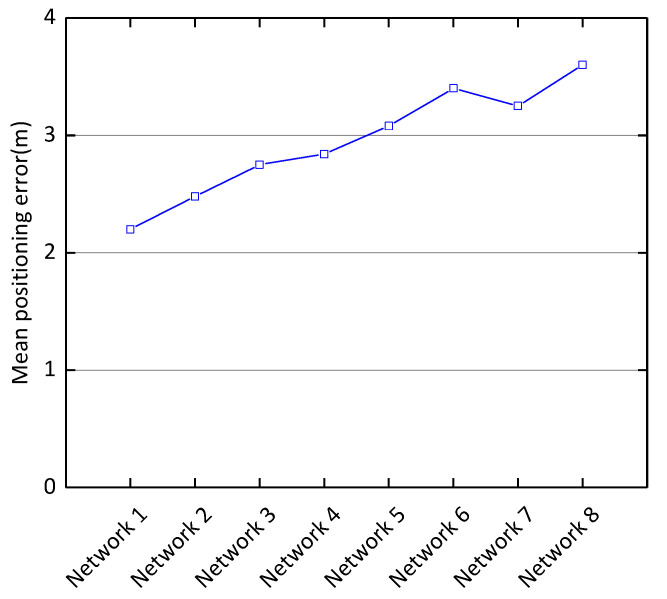
Mean positioning error of different network structures.

Effects of Network Structure on Positioning Performance. As shown in the Figure 4, we compare the mean positioning error for different layers, filters, and convolution kernels with a noise coefficient of 0.5. For example, CDAE (128–3, 64–3, max–2) + CNN (32–3, 16–3) means that the CDAE has two convolutional layers and one pooling layer, and the CNN has two convolutional layers. The first convolutional layer of CDAE has 128 output filters and a convolutional kernel size of 3; the second convolutional layer has 64 output filters with a convolutional kernel size of 3 and the maximum pooling window size is 2. The first convolutional layer of the CNN has 32 output filters with a convolutional kernel size of 3 and the second convolutional layer has 16 output filters with a convolutional kernel size of 3. Table 2 shows the different network structures compared experimentally and Figure 4 shows the different mean positioning error corresponding to the different network structures. The experimental result indicates that the structure with CDAE (140–2, 110–2, 90–2, max–2) + CNN (80–2, 60–2, max–2, 40–2, 20–2, max-2) can achieve a minimum mean positioning error of 2.2 m.

Effects of Dropout on Positioning Performance. To prevent overfitting of the neural network, two dropout layers, called dropout-layer-one and dropout-layer-two, are adopted. One dropout layer is before the CNN network convolution operation, and the other dropout layer is before the full connection. Dropout randomly selects a portion of weights not to be updated when backpropagation error is updated, which is equivalent to randomly deleting a portion of the hidden units (which are not actually deleted, but temporarily not used).

**Figure 5:**
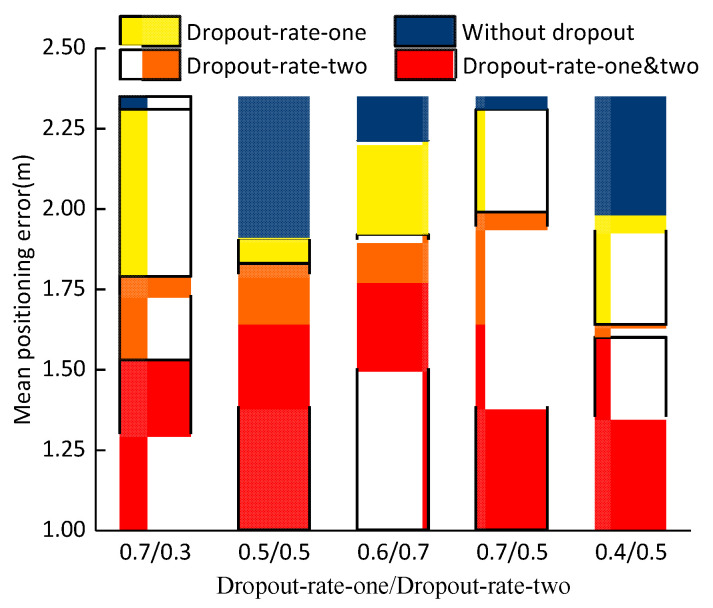
Effects of dropout on positioning performance.

Under the optimal network structure with a noise factor of 0.5, Figure 5 compares the effect of the presence of dropout layer-1 and dropout layer-2 on the mean positioning error. Additionally, we compare the effect of the two dropout layers’ rates on the positioning error.

The blue part represents the absence of the dropout layer. The yellow part represents the presence of dropout rate-one only. The orange part represents the presence of dropout rate-two only. And the red part represents the presence of both dropout rate-one and dropout rate-two. There are two numbers on the horizontal axis. The former number represents the value of dropout-rate-one, and the latter number represents the value of dropout-rate-two. It can be seen from the Figure 5 that when the dropout-layer-one and dropout-layer-two ratios are 0.7 and 0.3, respectively, the mean positioning error is the smallest and is 1.53 m.

**Figure 6:**
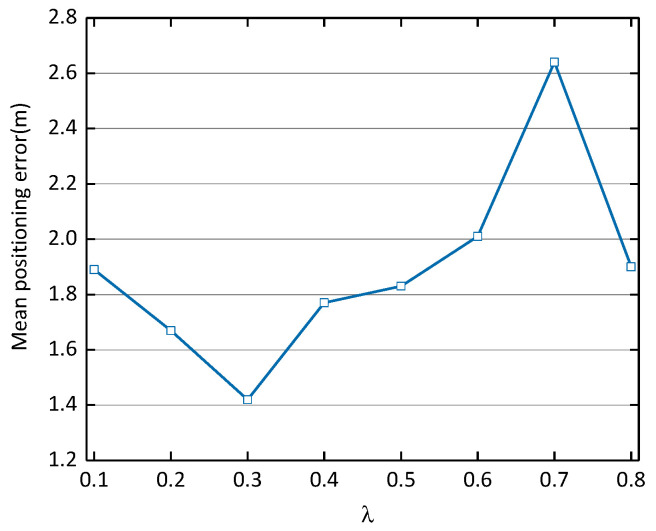
Effects of noise coefficient λ on positioning performance.

Effects of Noise Coefficient λ on Positioning Performance. In the case of the above optimal CDAE-CNN-one network structure and the optimal dropout rate, the mean positioning error obtained with different noise coefficients is compared. It can be seen from Figure 6 that the noise factor 0.3 achieves the best performance (achieving a mean positioning error of 1.42 m).

#### 4.2.2. Experiment of the K-Means Algorithm to Extract the Validation Set

When the individual parameters of the CDAE-CNN are as shown in Table 3, we proceed to verify the effect of the K-means algorithm on the average localization error according to Algorithm 1. As shown in Table 4, the mean positioning error is compared between k=110 and the randomly selected validation set, which has one-sixth of the total training set samples. We can see that the mean positioning error obtained at k = 110 is 1.18 m.

#### 4.2.3. Effect of RSSI Normalization Method on Average Positioning Error

This section is to obtain which of the three normalization methods (as shown in Equations (3)–(5)) for RSSI results in the smallest mean positioning error. We compare the mean positioning error obtained by each of the three methods as shown in Table 5, when the network parameters are as in Table 3 and k = 110.The previous experiments used the powed representation. According to Table 5, zero-to-one normalization method gives the best results with a mean positioning error of 1.05 m.

#### 4.2.4. Comparison Experiments with Other Methods

**Figure 7:**
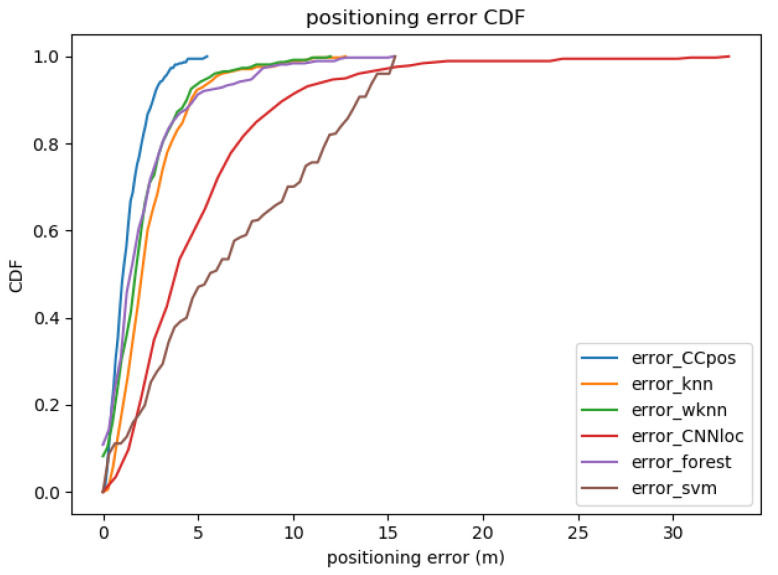
Positioning error with different methods on the Alcala Tutorial 2017 dataset.

The mean positioning errors obtained from the CCpos-based system and other fingerprint-based localization methods are compared as shown in Table 6. Figure 7 shows the cumulative distribution function (CDF) plot of the positioning error with the CCpos system and other fingerprint-based localization methods. We compare the KNN, the WKNN, SVM, random forests, and the CNNloc system [22]. The parameters of each compared method are shown in Table 7. From Table 6, it can be found that the mean positioning error of the CCpos system is at most 5.7 m and at least 1.2 m lower than that of other positioning methods. As can be seen from the CDF diagram in Figure 7, the possibility of positioning accuracy for CCpos better than 3 m is close to 98%. The possibility of positioning accuracy for WKNN, KNN and random forests algorithms better than 3 m is nearly 90%. The probability of CNNloc algorithm better than 3 m is about 50%, while the probability of SVM algorithm better than 3 m is only about 30%. It can be seen that the positioning accuracy of CCpos system is better than that of other positioning methods. 

**Figure 8:**
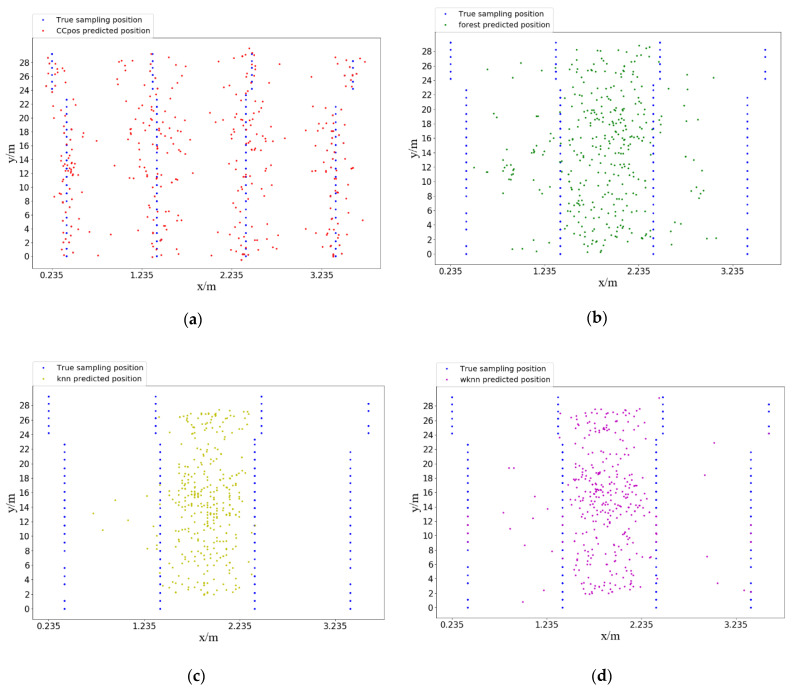
Comparison of true and predicted positions. (**a**) True position vs. CCpos predicted position; (**b**) true position vs. Random Forest predicted position. (**c**) true position vs. KNN algorithm predicted position; (**d**) true position vs. WKNN algorithm predicted position.

Figure 8 presents the predicted position compared with the true position in two-dimensional coordinates. Our test set contains 405 sample data and 103 sampling points. Each sampling point collects 3–4 RSSI data messages. Thus, there are 103 true positions (blue points) and 405 predicted positions (other color points). We compare the true positions and predicted positions of CCpos, Random Forest, KNN algorithm and WKNN algorithm respectively (as shown in Figure 8a–d), where the predicted positions obtained by CCpos system are closer to the true positions (blue points).

### 4.3. Evaluation Experiments on the UJIIndoorLoc Dataset 

This subsection presents the optimization of the system model based on the UJIIndoorLoc dataset and the comparison of the experimental results with other WiFi fingerprint-based methods. Since the UJIIndoorLoc dataset covers three buildings of 4–5 floors, floor prediction model and building prediction model are added on the basis of location prediction model.

The best performance of the system can be achieved (the floor location accuracy is 95.3%, the building location accuracy is 99.6%, and the mean positioning error is 12.4 m) when the model parameters are shown in Table 8, the K-mean algorithm has the clustering center K = 2000, and the RSSI uses the zero-to-one normalization method. Among them, a dropout layer is added after the CDAE coding layer, and a dropout layer is added before the fully connected layer of each model. The respective dropout rates are shown in the following table. The loss function used in each network structure is the mean square error (MSE). The GWN factor of CDAE is 0.3, and the patience parameter *X* of Early Stopping method is 3.

Table 9 compares the mean positioning error of using K-means to extract the validation set and randomly selected validation set. It can be found that the K-means algorithm to extract the validation set is more effective on small datasets.

**Figure 9:**
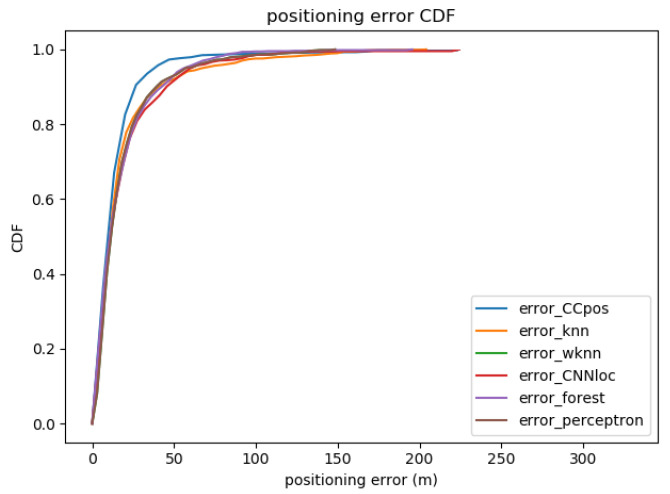
Positioning error with different methods on the UJIIndoorLoc dataset.

Table 10 compares the mean positioning error obtained on the UJIIndoorLoc dataset between the CCpos-based system and other fingerprint-based localization methods. Figure 9 shows the CDF plot of the positioning error for each positioning method. We compare the KNN, WKNN, gradient boosting algorithm (gradient), random forest, and CNNloc system. The parameters of each algorithm are shown in Table 11. It can be found that the CCpos system reduces the average localization error by up to 33% and at least 22% compared to other localization algorithms. The CDF plot of the positioning error in Figure 9 shows that the possibility of positioning accuracy for CCpos better than 30 m is more than 90%, while the possibility of positioning accuracy for other localization algorithms better than 30 m is around 80%. 

## 5. Conclusions

In this paper, we propose the CCpos positioning system, a WiFi fingerprint positioning system based on CDAE and CNN. CDAE can denoise and extract features from RSSI data, which effectively improves the accuracy of CNN for location prediction. The system applies the K-means localization algorithm to extract the validation set, and the experiment proves that the method has better results for localization of small datasets. In this paper, the system is evaluated on two datasets, UJIIndoorLoc and Alcala Tutorial 2017 Data Set, and the CCpos system obtains the smallest mean positioning error compared to other fingerprint-based localization methods (UJIIndoorLoc and Alcala Tutorial 2017 dataset have mean positioning errors of 12.4 m and 1.05 m, respectively). The experimental results prove that CCpos system is more advantageous in small positioning scenarios and can also achieve smaller positioning errors in large positioning ranges. Without other positioning devices, WiFi positioning is convenient and efficient. However, the drawback of this system is that it needs to be tuned according to different scenarios in practical application. Also, due to the limited positioning accuracy that WiFi positioning can achieve, the next step in the research is to combine WiFi positioning with other sensors (such as IMU, Bluetooth, etc.) positioning to improve positioning accuracy.

## Figures and Tables

**Table 1:** Model parameter.

Parameter	Values
GWN factor	λ
CDAE activation function	ReLU
CDAE Optimizer	Nadam
CDAE Loss	MSE
CDAE metrics	MAE
CNN activation function	ReLU
CNN Optimizer	Nadam
CNN loss	MSE
CDAE metrics	MAE
Output layer activation function	ReLU
Batch size	18
Early Stopping patience *X*	4

**Table 2:** Network structure and its sequence number.

Network Structures	Serial Number
CDAE (140–2, 110–2, 90–2, max–2) +CNN (80–2, 60–2, max–2, 40–2, 20–2, max–2)	Network 1
CDAE (140–2, 100–2, 80–2, max–2) +CNN (80–2, 60–2, max–2, 40–2, 20–2, max–2)	Network 2
CDAE (140–2, 100–2, 90–2, max–2) +CNN (80–2, 60–2, max–2, 40–2, max–2)	Network 3
CDAE (140–2, 100–2, 80–2, max–2) +CNN (80–2, 60–2, max–2)	Network 4
CDAE (140–2, 100–2, max–2) +CNN (80–2, 60–2, max–2)	Network5
CDAE (140–2, 100–2, max–2) +CNN (100–2, 80–2)	Network 6
CDAE (140–2, 100–2, max–2) +CNN (80–2, 60–2)	Network 7
CDAE (128–3, 64–3, max–2) +CNN (32–3, 16–3)	Network 8

**Table 3:** CDAE-CNN optimal parameter.

Network Structure	Noise Coefficient λ	Dropout-One	Dropout-Two
CDAE (140–2, 110–2, 90–2, max–2) + CNN (80–2, 60–2, max–2, 40–2, 20–2, max–2)	0.3	0.7	0.3

**Table 4:** Comparison of validation sets obtained by different methods on the Alcala Tutorial 2017 dataset.

	K-means (K = 110)	Random
Mean positioning error (m)	1.18	1.42

**Table 5:** Comparison of different Normalization method on the Alcala Tutorial 2017 dataset.

	Zero-to-One Normalized	Exponential	Powed
mean positioning error (m)	1.05	4.5	1.18

**Table 6:** Mean positioning error with different methods on the Alcala Tutorial 2017 dataset.

Positioning Methods	Mean Positioning Error (m)
KNN	2.62
WKNN	2.27
SVM	6.71
Random forest	2.53
CNNloc	4.62
CCpos	1.05

**Table 7:** The algorithms and their parameters.

Positioning Method	Parameter
KNN	n_neighbors = 40
WKNN	n_neighbors = 30
SVM	C=1000.gamma = 0.01
Random forest	n_estimators = 120
CNNloc	SAE (128-64-128) + CNN (99-22,66-22,33-22)

**Table 8:** Model parameters on the UJIIndoorLoc dataset.

		Network	Dropout Rate	Optimizer	Batch Size	Activation Function	Output Layer Activation Function	Monitoring Metrics
CDAE		454–33, 388–33, 256–33	0.7	Adam (lr = 0.0001)	70	relu	relu	mae
CNN	Building- model	64–33, 31–22, max–2, 32–22, 16–11, max–2	0.5	Adam (lr = 0.0001)	70	relu	softmax	accuracy
	Floor- model	256–33, 128–33, max–2, 128–33, 64–33, max–2, 64–22, 32–11, max–2, 32–11, 16–11, max–2	0.3	Adam (lr = 0.0001)	70	relu	softmax	accuracy
	Positioning-model	256–33, 128–33, max–2, 64–33, 32–22, max–2, 32–22, 16–11, max–2	0.3	Adam (lr = 0.0001)	70	relu	relu	mae

**Table 9:** Comparison of validation sets obtained by different methods on the UJIIndoorLoc dataset.

	K-means (K = 110)	Random
Mean positioning error (m)	12.4	13.1

**Table 10:** Mean positioning error with different methods on the UJIIndoorLoc dataset.

Positioning Methods	Mean Positioning Error (m)
KNN	18.6
WKNN	18.4
gradient	18.5
random forests	15.9
CNNloc	16.9
CCpos	12.4

**Table 11:** The algorithms and their parameters.

Positioning Method	Parameter
KNN	n_neighbors = 10
WKNN	n_neighbors = 30
gradient	n_estimators = 100, max_depth = 10
random forests	n_estimators = 150
CNNloc	SAE (128–64–128) + CNN (99–22, 66–22, 33–22)

## Data Availability

Data is contained within the article.
